# A genome-wide scan of selective sweeps in two broiler chicken lines divergently selected for abdominal fat content

**DOI:** 10.1186/1471-2164-13-704

**Published:** 2012-12-15

**Authors:** Hui Zhang, Shou-Zhi Wang, Zhi-Peng Wang, Yang Da, Ning Wang, Xiao-Xiang Hu, Yuan-Dan Zhang, Yu-Xiang Wang, Li Leng, Zhi-Quan Tang, Hui Li

**Affiliations:** 1 Key Laboratory of Chicken Genetics and Breeding, Ministry of Agriculture, Harbin, 150030, P.R. China; 2 College of Animal Science and Technology, Northeast Agricultural University, Harbin, 150030, P.R. China; 3 Department of Animal Science, University of Minnesota, Saint Paul, Minnesota, 55108, USA; 4 College of Biological Science, China Agricultural University, Beijing, 100193, P.R. China; 5 Animal Genetics and Breeding Unit, University of New England, Armidale, New South Wales, 2351, Australia

**Keywords:** Abdominal fat, Selection signature, Extended haplotype homozygosity (EHH)

## Abstract

**Background:**

Genomic regions controlling abdominal fatness (AF) were studied in the Northeast Agricultural University broiler line divergently selected for AF. In this study, the chicken 60KSNP chip and extended haplotype homozygosity (EHH) test were used to detect genome-wide signatures of AF.

**Results:**

A total of 5357 and 5593 core regions were detected in the lean and fat lines, and 51 and 57 reached a significant level (*P*<0.01), respectively. A number of genes in the significant core regions, including *RB1*, *BBS7*, *MAOA*, *MAOB*, *EHBP1*, *LRP2BP*, *LRP1B*, *MYO7A*, *MYO9A* and *PRPSAP1*, were detected. These genes may be important for AF deposition in chickens.

**Conclusions:**

We provide a genome-wide map of selection signatures in the chicken genome, and make a contribution to the better understanding the mechanisms of selection for AF content in chickens. The selection for low AF in commercial breeding using this information will accelerate the breeding progress.

## Background

The linkage disequilibrium (LD) is important in livestock genetics for its key role in genomic selection [1] and detecting the causal mutations of economically important traits [2-6]. Based on the LD information, there are two main methods to detect genes underlying phenotypic variation, including one from phenotype to genome and another one from genome to phenotype. The first method is performed by targeting particular candidate genes or by quantitative trait loci (QTL) mapping and positional cloning of QTL. In the second method, patterns of LD in populations that are incompatible with the hypothesis of genetic neutrality are identified, and these patterns are selection signatures [7]. The aim of the second method is to identify artificial selections by statistically evaluating the genomic data [7].

Allele frequencies underlying selection are expected to change. A neutral mutation will take many generations until the mutated allele reaches a high or low population frequency. In this case, the LD between the mutation and its neighboring loci will be degraded because of the recombination in every generation [8]. The frequency of a novel mutation will increase or decrease more rapidly than the neutral mutation because it is underlying artificial selection, so that the surrounding conserved haplotype was long [9,10]. This is the background of the extended haplotype homozygosity (EHH) statistic method used to detect selection signatures [11]. There are also many other methods to detect selective sweeps from DNA sequence data, including the Tajima’s *D*[12] and Fay and Wu’s *H*-test [13] for selected mutations, measuring large allele-frequency differences among populations by F_ST_[14], and the integrated Haplotype Score (iHS) [15], which is an extension of the EHH statistic [11]. Among these methods, the EHH test is particularly useful [7,11]. The EHH test is used to detect artificial selections according to the characteristics of haplotypes within a single population, and do not require the genotype of the ancestor [7]. Furthermore, the EHH test is less sensitive to ascertainment bias than other approaches, so it was designed to work with SNP rather than sequencing data [7,16].

The broilers used in this study were selected for eleven generations and genomic regions controlling AF deposition are expected to exhibit signatures of selective sweep. The aim of this study was to identify the selection signatures underlying the artificial selection for AF in chicken and to investigate the genes important for AF deposition.

## Methods

### Ethics statement

All animal work was conducted according to the guidelines for the care and use of experimental animals established by the Ministry of Science and Technology of the People’s Republic of China (Approval number: 2006–398) and approved by the Laboratory Animal Management Committee of Northeast Agricultural University.

### DNA samples and data preparation

**Figure 1:**
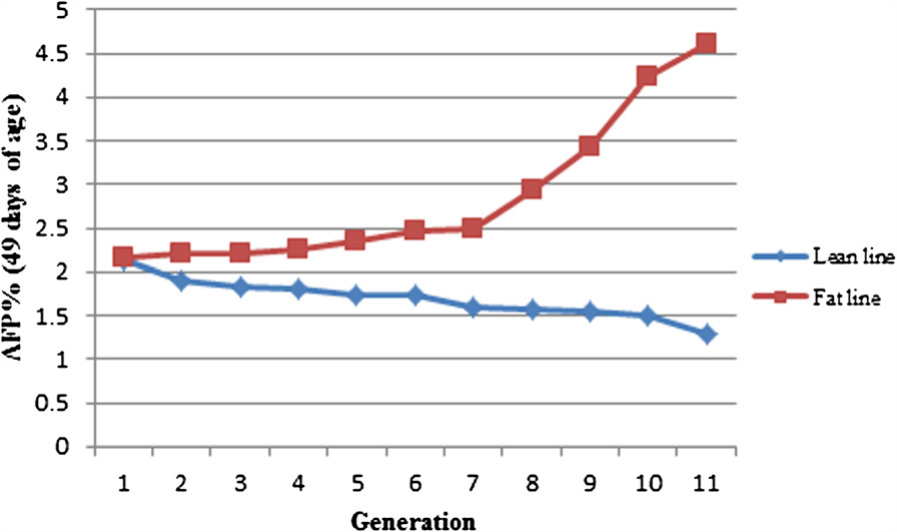
The separation of AFP over 11 generations between lean and fat lines.

Broilers used in this study were from two Northeast Agricultural University broiler lines divergently selected for AF content (NEAUHLF). The two lines have been selected since 1996 using AF percentage (%AFW or AFP) and plasma very low-density lipoprotein (VLDL) concentration as selection criteria [17]. The two lines were selected for 11 generations and the AFP changes over the 11 generations are shown in Figure 1. A total of 475 individuals from generation 11 of NEAUHLF were used in this study.

Genotyping was carried out using the Illumina chicken 60K SNP chip containing a total of 57636 SNPs. Markers were filtered to exclude loci with unknown positions, monomorphic loci and loci with a minor allele frequency <0.05.

### The haplotype and LD analysis

The fastPHASE [18] (http://depts.washington.edu/fphase/download/) was used to reconstruct the haplotypes for every chromosome using the default parameters. The reconstructed haplotypes were inserted into HAPLOVIEW v4.1 [19] to estimate LD statistics based on pairwise r^2^ and to construct the blocking pattern in the candidate regions of interest to enable selection signature analysis.

### The EHH test

The “core region” was defined as the region in the genome characterized by the strong LD among SNPs involving a set of “core haplotypes” [7]. The Sweep v.1.1 (http://www.soft82.com/get/download/windows/sweep/) was used to identify the core regions [11]. The algorithm defined a pair of SNPs to be in strong LD if the upper 95% confidence bound of D’ is between 0.70 and 0.98 [20]. The program was set to select core regions with at least two SNPs. EHH was defined as the probability that two randomly chosen haplotypes carrying the candidate core haplotype were homozygous for the entire interval spanning the core region to a given locus [11]. The EHH test [11] was based on one of the core haplotype vs. other haplotypes in the same position. The “Relative Extended Haplotype Homozygosity” (REHH) statistic corrects EHH for the variability in recombination rates [7]. It was computed by EHH_t_ / $\bar{\mathrm{EHH}}$; with $\bar{\mathrm{EHH}}$ defined as the decay of EHH on all other core haplotypes combined. The REHH value was used in the current study to determine the selection signatures. To determine the significance of REHH values, the haplotypes were ordered into 20 bins according to their frequencies [7]. The REHH values of each haplotype in a candidate region were compared with all equally frequent haplotypes and the *P*-values were obtained [11]. The significant selection signatures were defined as *P*<0.01.

## Results

### Markers and core haplotypes

A total of 43034 SNPs on 28 autosomes in chickens were included in the selection signature analysis (Table 1). These markers covered 950.68 Mb of the genome, with an average of 22.09 kb between adjacent markers.

**Table 1:** Summary of genome-wide marker and core region (CR) distribution in the lean and fat lines

**Chr**	**SNP (n)^1^**	**Chr length (Mbp)**	**Mean distance (kb)**	**No. CR (n)**		**Mean CR length (kb)**		**Coverage CR length^2^ (kb)**		**Max CR length (kb)**		**CR length/Chr length^3^**		**CR SNPs^4^ (n)**		**Max CR SNPs (n)**		**CR SNPs/SNP^5^**	
				**Lean line**	**Fat line**	**Lean line**	**Fat line**	**Lean line**	**Fat line**	**Lean line**	**Fat line**	**Lean line**	**Fat line**	**Lean line**	**Fat line**	**Lean line**	**Fat line**	**Lean line**	**Fat line**
1	7135	200.95	28.16	881	920	125.59	114.92	110644.43	105728.03	2288.64	2191.34	0.55	0.53	3906	3716	19	19	0.55	0.52
2	5290	154.46	29.20	639	695	149.62	108.91	95606.56	75690.16	2048.43	2042.96	0.62	0.49	3260	2628	19	19	0.62	0.50
3	4081	113.65	27.85	517	533	121.58	108.97	62855.68	58081.43	863.98	735.27	0.55	0.51	2301	2107	19	19	0.56	0.52
4	3313	94.16	28.42	411	428	137.96	108.07	56701.29	46255.07	2087.33	611.37	0.60	0.49	1992	1676	19	19	0.60	0.51
5	2170	62.23	28.68	260	266	138.85	105.39	36101.29	28034.75	823.62	816.35	0.58	0.45	1282	1032	19	19	0.59	0.48
6	1714	35.84	20.91	217	225	94.92	72.79	20598.01	16377.61	535.90	523.04	0.57	0.46	983	826	19	19	0.57	0.48
7	1769	38.17	21.58	197	232	111.15	86.03	21897.27	19958.16	621.29	2163.72	0.57	0.52	1048	899	19	19	0.59	0.51
8	1394	30.62	21.97	159	175	111.56	96.82	17738.07	16944.10	1914.74	1949.21	0.58	0.55	791	763	19	19	0.57	0.55
9	1168	24.02	20.57	159	153	78.35	75.92	12457.00	11615.65	413.33	403.29	0.52	0.48	613	557	19	17	0.52	0.48
10	1297	22.42	17.29	172	176	70.99	63.35	12210.13	11148.99	387.48	347.35	0.54	0.50	735	699	19	19	0.57	0.54
11	1196	21.87	18.29	128	156	124.72	83.15	15964.06	12971.74	886.96	1093.97	0.73	0.59	871	706	19	19	0.73	0.59
12	1324	20.45	15.44	169	184	71.34	51.16	12057.10	9412.86	352.96	369.92	0.59	0.46	809	633	19	19	0.61	0.48
13	1128	18.32	16.24	144	141	75.86	75.09	10924.53	10584.67	373.56	373.56	0.60	0.58	695	656	19	19	0.62	0.58
14	984	15.76	16.02	127	123	75.17	68.77	9546.48	8459.25	402.70	402.70	0.61	0.54	598	544	19	19	0.61	0.55
15	1010	12.93	12.80	123	133	58.20	50.28	7158.60	6687.12	407.05	407.05	0.55	0.52	567	541	19	19	0.56	0.54
16	12	0.17	13.87	3	1	41.85	67.25	125.54	67.25	64.36	67.25	0.74	0.40	9	3	4	3	0.75	0.25
17	844	10.61	12.57	112	108	59.07	43.89	6616.05	4740.59	242.32	236.98	0.62	0.45	523	394	19	19	0.62	0.47
18	845	10.89	12.88	112	121	48.74	45.96	5459.42	5561.31	317.30	317.30	0.50	0.51	431	431	12	19	0.51	0.51
19	804	9.89	12.31	117	110	36.01	48.41	4212.67	5325.00	406.27	371.08	0.43	0.54	353	421	14	19	0.44	0.52
20	1460	13.92	9.53	184	181	45.89	46.33	8442.96	8386.18	273.60	270.67	0.61	0.60	904	888	19	19	0.62	0.61
21	726	6.88	9.47	81	90	47.74	35.17	3867.13	3165.72	211.67	196.05	0.56	0.46	432	354	19	18	0.60	0.49
22	295	3.89	13.19	36	30	71.16	79.29	2561.59	2378.83	267.88	289.01	0.66	0.61	193	182	19	19	0.65	0.62
23	577	6.02	10.44	81	80	37.13	31.74	3007.73	2539.51	239.20	239.20	0.50	0.42	307	272	19	19	0.53	0.47
24	676	6.23	9.22	87	91	40.77	32.96	3546.91	2999.26	133.00	212.48	0.57	0.48	387	339	13	19	0.57	0.50
25	170	2.02	11.86	23	18	34.97	32.38	804.26	582.82	82.74	72.39	0.40	0.29	99	68	12	10	0.58	0.40
26	617	5.03	8.16	81	85	34.55	59.60	2798.60	2515.94	246.20	278.91	0.56	0.50	345	312	19	19	0.56	0.51
27	472	4.84	10.25	60	59	46.66	40.46	2799.62	2387.38	384.65	482.24	0.58	0.49	299	215	19	19	0.63	0.46
28	563	4.46	7.92	77	79	36.64	27.66	2820.93	2185.41	520.13	172.85	0.63	0.49	336	318	19	19	0.60	0.56
Total	43034	950.68	22.09	5357	5593	102.58	85.96	549523.91	480784.79	2288.64	2191.34	0.58	0.51	25069	22180	19	19	0.58	0.52

^1^The number of SNPs.

^2^Total length covered by core regions.

^3^The proportion of total core region lengths on chromosome length.

^4^Number of SNPs in core regions.

^5^The proportion of total number of SNPs in core regions on number of SNPs used.

**Figure 2:**
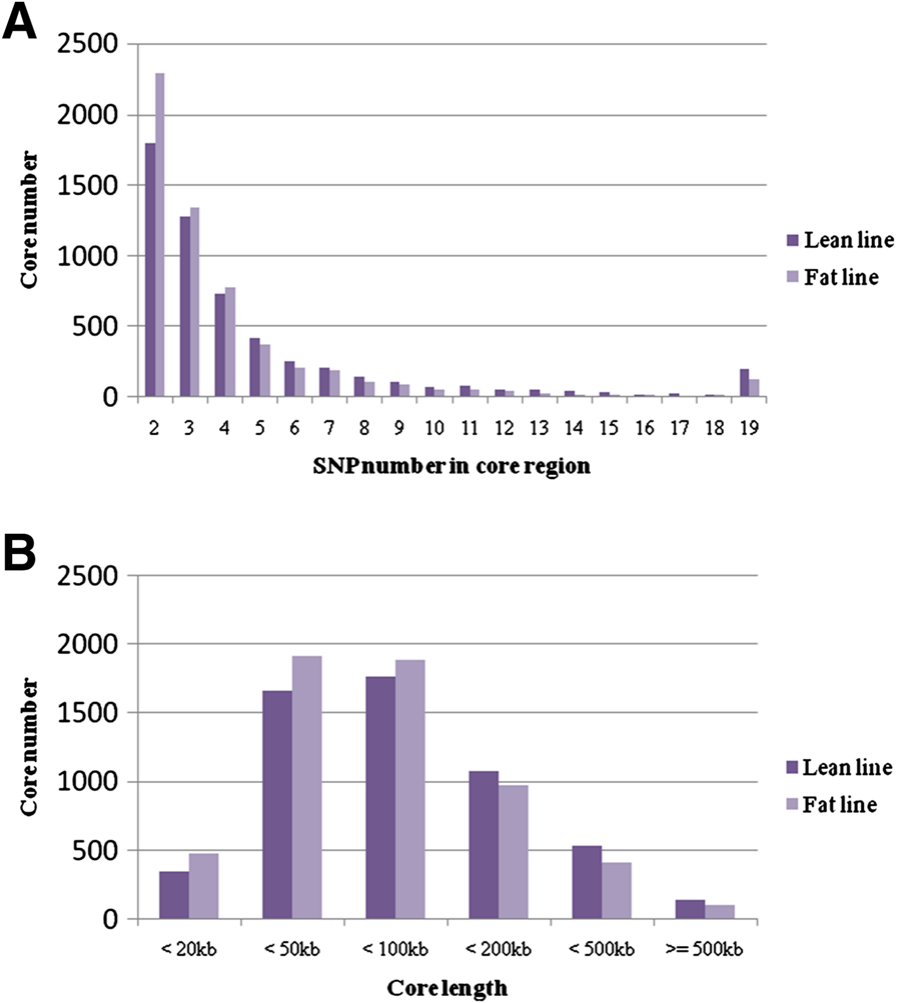
Distribution of SNP numbers in the core regions (A) and the length of core regions (B) in lean and fat lines.

For the SNPs analyzed in this study, the average minor allele frequency was 0.29 ± 0.13. A summary of genome-wide markers and core haplotype distribution in the data set is shown in Table 1. A total of 5357 and 5593 core regions spanning 549523.91 kb and 480784.79 kb of the genome, respectively, in the lean and fat lines were detected (Table 1). Mean core region length was estimated as 102.58±37.24 kb and 85.96±26.65 kb, with a maximum of 2288.64 kb and 2191.34 kb in the lean and fat lines, respectively (Table 1). Chromosome 1 was the largest chromosome in chickens, and it had the largest haplotypic structures in the genome, which covered 110644.43 kb and 105728.03 kb in the lean and fat lines, respectively. For each chromosome, the proportion of length covered by core regions vs. total length, as well as the number of SNPs forming core regions vs. the total number of SNPs, are shown in Table 1. The distribution of the size of core regions is shown in Figure 2. Overall, 25069 and 22180 SNPs in the lean and fat lines, respectively, participated in forming core regions, with a range of 2 to 19 SNPs per core.

### Whole genome selection signatures

**Figure 3:**
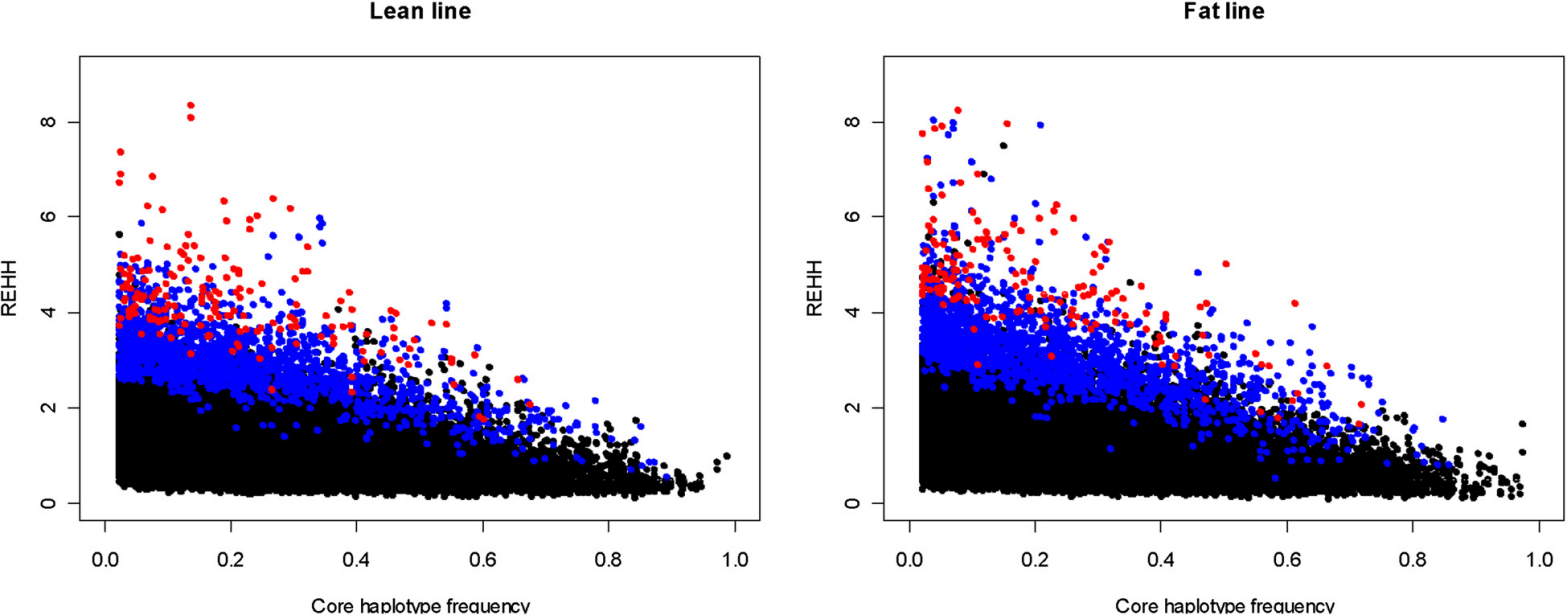
**Distribution of REHH vs. core haplotype frequencies in the lean and fat lines.** Core haplotypes with P-values lower than 0.05 and 0.01 are presented in blue and red, respectively.

**Figure 4:**
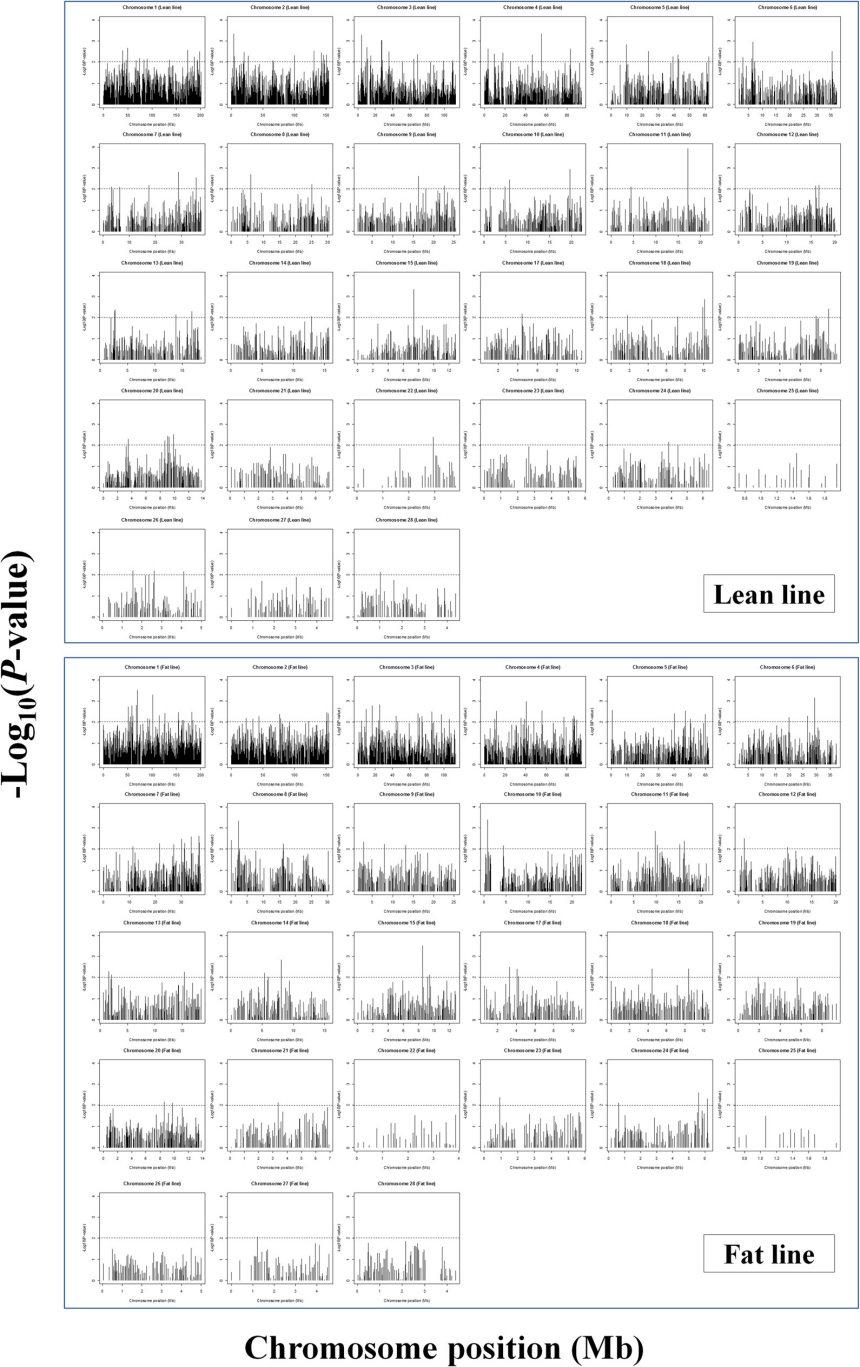
**Genome-wide map of *P*-values for core haplotypes with frequency >0.25 in lean and fat lines, respectively.** Dashed lines display the threshold level of 0.01.

For all 5357 and 5593 core regions in the lean and fat lines, respectively, a total of 44822 and 46775 EHH tests, with an average of 8.37 and 8.36 tests per core region, were calculated. To find outlying core haplotypes, we calculated REHH at 1 Mb distances both on the upstream and downstream sides. Figure 3 shows the distribution of REHH values vs. haplotype frequencies in the lean and fat lines, respectively. Corresponding *P*-values are indicated by different colored symbols. The –log_10_ of the *P*-values associated with REHH against the chromosomal position was plotted to visualize the chromosomal distribution of outlying core haplotypes with frequency <25% (Figure 4). The results indicated that these selection signals were not uniformly distributed across all chromosomes, with a substantial overrepresentation on chromosomes 1, 2, 3 and 4.

The genome-wide statistics of the selection signature test, including the number of tests and outlying core haplotypes for each chromosome, are presented in Table 2. Of 16677 and 18346 tests on core haplotypes with frequency ≥0.25, there were 51 and 57 tests with *P*<0.01 in the lean and fat lines, respectively. There were 153 and 251 tests with *P*<0.05 in the lean and fat lines, respectively.

**Table 2:** **The number of tests on core haplotypes (CH) (both sides) with frequency≥0.25 and *P*-values of REHH test**

**Chr**	**Lean line**			**Fat line**		
	**Test on CH**	***P*-value <0.05**	***P*-value <0.01**	**Test on CH**	***P*-value <0.05**	***P*-value <0.01**
1	2806	113	4	3063	138	12
2	2009	105	8	2271	104	3
3	1654	79	10	1705	74	8
4	1273	58	6	1371	66	5
5	844	34	3	883	36	4
6	699	25	2	757	31	2
7	638	29	2	770	31	5
8	464	16	1	574	33	2
9	516	20	1	564	19	1
10	540	23	2	582	27	1
11	397	15	1	534	20	2
12	503	14	0	619	20	1
13	447	19	3	474	22	1
14	379	14	0	418	16	1
15	329	12	1	420	18	2
17	350	16	0	348	14	2
18	354	12	2	432	13	2
19	334	12	1	338	13	0
20	561	28	3	566	19	0
21	255	6	0	304	11	0
22	105	5	1	85	2	0
23	258	11	0	245	12	1
24	287	9	0	308	12	2
25	46	1	0	36	1	0
26	231	11	0	253	4	0
27	181	8	0	184	7	0
28	217	5	0	242	14	0
Total	16,677	700	51	18,346	777	57

**Figure 5:**
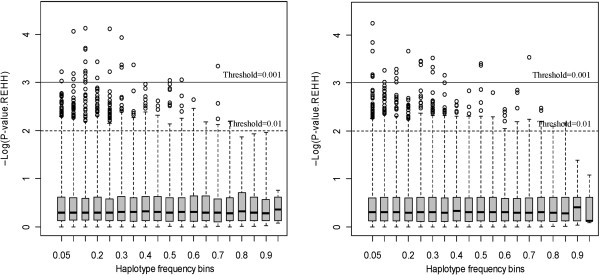
**Box plot of the distribution of *P*-values in core haplotype frequency bins in the lean (left) and fat (right) lines.** The dashed and continuous lines indicated the threshold *P*-values of 0.01 and 0.001, respectively.

The conformity of the distribution of Tukey’s outliers was examined, with outlying core haplotypes defined at the threshold level of 0.01. Figure 5 displays box plots of the distribution of –log10 (*P*-values) within each bin of core haplotype frequency. The results indicated that the extreme outliers appear in the small haplotype frequencies bins.

### Mapping selection signatures to genes

A summary of statistics for 51 and 57 positively selected core regions with *P*<0.01 of the REHH tests in the lean and fat lines, respectively, is presented in Table 3. Corresponding genes were identified by aligning the core positions with the chicken genome sequence (Table 3). The full genes names were from Ensembl online (http://www.ensembl.org/index.html). A total of 66 and 46 genes in the core regions were detected in the lean and fat lines, respectively, including *RB1* (retinoblastoma 1), *BBS7* (Bardet-Biedl syndrome 7), *MAOA* (monoamine oxidase A), *MAOB* (monoamine oxidase B), *EHBP1* (EH domain binding protein 1), *LRP2BP* (LRP2 binding protein), *LRP1B* (low-density lipoprotein receptor-related protein 1B), *MYO7A* (myosin VIIA), *MYO9A* (myosin IXA) and *PRPSAP1* (phosphoribosyl pyrophosphate synthetase-associated protein 1). The haplotype analysis of these genes revealed that the haplotype frequencies were significantly different (*P*<0.01) between the two lines (Table 4).

**Table 3:** **Statistics summary for core haplotypes with *P*<0.01 after the relative extended haplotype homozygosity (REHH) test**

**Lean line**						
**Chr**	**Core position**	**Hap Freq**	**EHH**	**REHH^1^**	**REHH *P*-value^1^**	**Genes**
1	39360501-39455853	0.46	0.98	3.99	0.0027	/
1	49926970-49964278	0.30	0.97	4.18	0.0021	*C12orf69*, *WBP11*, *H2A4*, *H2B1*, *H4*, *H32*, *H2B8*
1	173098805-173190831	0.37	0.99	4.25	0.0027	***RB1***, *LPAR6*, *O57531*, *RCBTB2*
1	198071099-198113519	0.55	0.80	3.03	0.0031	*GDPD4*, ***MYO7A***
2	3631683-3739002	0.32	0.70	4.88	0.0004	*Q5ZK34*
2	19934135-20028093	0.30	0.97	3.88	0.0035	*RSU1*
2	26912546-26974875	0.28	1.00	3.65	0.0050	/
2	99818321-100051643	0.41	1.00	3.00	0.0047	*GNAL*, *NRGN*
2	131104507-131150076	0.48	0.98	3.24	0.0029	*Q6V0P0*, *INTS8*, *F1P3N8*
2	143016981-143059231	0.36	0.97	3.46	0.0034	/
2	145836411-145908271	0.30	1.00	3.72	0.0045	/
2	150489129-150540434	0.34	1.00	3.51	0.0044	/
3	3794973-3861882	0.30	0.79	4.72	0.0005	*C20orf26*, *CRNKL1*
3	3794973-3861882	0.30	0.82	3.85	0.0020	*C20orf26*, *CRNKL1*
3	10257926-10454969	0.55	0.49	2.49	0.0019	*F1NRN6*
3	14895290-14957057	0.44	0.72	3.16	0.0048	*PLCB4*
3	26957549-26996618	0.46	0.80	3.69	0.0009	/
3	26957549-26996618	0.46	0.83	3.47	0.0013	/
3	27303800-27335510	0.52	0.92	3.78	0.0009	*SRBD1*
3	27382993-27430067	0.54	0.84	3.77	0.0009	*SRBD1*
3	35555718-35610466	0.47	0.66	3.02	0.0031	*E1C4G2*
3	68936320-69076223	0.27	0.98	3.79	0.0041	*RPF2*, *GTF3C6*, *Q5F484*, *CDK19*
4	3522359-3551494	0.59	0.54	3.10	0.0023	*MBNL3*
4	9568761-9604871	0.55	1.00	2.95	0.0040	/
4	17765695-17819334	0.41	1.00	3.56	0.0037	*F1NEF4*, *HMGB3*
4	46149116-46190279	0.36	0.99	3.35	0.0044	*EREG*, *Q645M5*
4	55424480-55472209	0.66	0.9	2.60	0.0005	*TRPC3*, ***BBS7***
4	83051637-83117974	0.39	0.87	3.72	0.0022	/
5	9740941-9828144	0.49	1.00	3.42	0.0014	*IF4G2*, *CTR9*, *MRVI1*
5	23825115-23872187	0.41	0.78	3.19	0.0030	*O93582*
5	42592517-42679460	0.30	1.00	3.70	0.0044	/
6	6546601-6626145	0.39	1.00	4.42	0.0011	/
6	35354459-35390346	0.38	0.99	3.72	0.0030	*PTPRE*
7	28869664-28906344	0.31	1.00	4.88	0.0015	*MYLK*
7	35674098-35715122	0.67	0.48	2.09	0.0027	
8	6107407-6172105	0.36	0.63	3.72	0.0020	*IER5*, *KIAA1614*, *XPR1*
9	16264832-16366749	0.45	0.97	4.04	0.0024	*PSMD1*, *ARMC9*, *B3GNT7*
10	5831963-5856349	0.59	0.97	1.83	0.0034	/
10	19717086-19745274	0.48	1.00	2.90	0.0011	*CHSY1*
11	17094961-17160195	0.30	0.63	3.35	0.0001	*BCDO1*, *GAN*
13	2628777-2664596	0.35	1.00	3.94	0.0048	*Q5ZHQ9*
13	2726706-2746894	0.39	0.79	4.06	0.0041	/
13	16758621-16783127	0.26	0.69	3.26	0.0050	*FSTL4*
15	7345639-7377799	0.26	0.54	2.38	0.0004	*SEZ6L*, *ASPHD2*, *HPS4*
18	9949736-10015444	0.39	1.00	2.34	0.0031	*SPAG9*
18	10117401-10135964	0.39	1.00	2.64	0.0013	*F1NM51*
19	8727596-8786448	0.60	0.55	1.77	0.0038	*MSI1*
20	9090808-9113453	0.29	0.95	6.18	0.0036	*MYT1*
20	9246245-9278998	0.32	0.80	5.39	0.0040	*E1C8M0*
20	9879361-9899719	0.27	0.96	6.40	0.0030	*CSK21*
22	2952274-3002268	0.29	0.29	3.93	0.0039	/
Fat line						
1	51248496-51279543	0.33	0.81	4.45	0.0018	*TCF20*
1	58120009-58215364	0.55	0.99	3.15	0.0016	*Q8UVD4*
1	60171076-60254771	0.46	1.00	4.12	0.0049	/
1	67763862-67830818	0.26	0.96	4.06	0.0026	/
1	68213617-68257241	0.61	0.94	4.21	0.0016	*SOX5*
1	69634186-69686357	0.66	0.99	2.89	0.0003	/
1	101535615-101635667	0.29	1.00	5.24	0.0005	*SAMSN1*
1	114789487-114875623	0.29	0.99	3.66	0.0048	***MAOB***, ***MAOA***
1	125909995-126011984	0.35	1.00	3.77	0.0036	*E1BTB5*
1	154665510-154752965	0.72	0.89	2.09	0.0034	/
1	181800227-181883545	0.33	0.99	4.00	0.0033	*A1XGV6*
1	181800227-181883545	0.33	1.00	3.81	0.0043	*A1XGV6*
2	76768841-76854523	0.31	1.00	4.30	0.0041	/
2	151203953-151251059	0.62	0.82	2.32	0.0033	*TRAPPC9*
2	153117092-153143883	0.71	0.77	1.68	0.0038	/
3	9177907-9222825	0.38	0.95	4.00	0.0024	***EHBP1***
3	9177907-9222825	0.38	0.90	3.66	0.0039	***EHBP1***
3	16143474-16194865	0.30	0.98	4.98	0.0016	/
3	24945839-24986772	0.61	0.70	2.15	0.0014	/
3	44265116-44311493	0.40	0.99	3.50	0.0050	*UNC93A*
3	69863850-69906698	0.34	1.00	4.29	0.0038	/
3	85874137-85931473	0.41	1.00	3.97	0.0031	*LMBRD1*
3	97227680-97337906	0.28	0.99	3.81	0.0042	/
4	11582141-11642538	0.27	0.96	4.53	0.0029	/
4	40653593-40713404	0.32	0.93	4.40	0.0010	*C4orf20*, ***LRP2BP***, *SNX25*
4	55950677-55991394	0.28	0.99	4.55	0.0028	/
4	55950677-55991394	0.28	1.00	4.36	0.0036	/
4	86719441-86754976	0.57	0.75	2.88	0.0048	/
5	556571-628531	0.25	0.67	4.37	0.0026	*F1NYX6*, *PLCB2*, *BUB1B*, *PAK6*
5	40239840-40261525	0.29	0.97	4.48	0.0038	*VSX2*, *F1N9P5*
5	47240577-47282933	0.40	0.95	2.91	0.0029	*RIN3*, *LGMN*
5	59811459-59880511	0.39	0.66	3.36	0.0041	/
6	26756202-26793956	0.34	0.98	3.94	0.0049	/
6	29341938-29401207	0.32	0.97	5.50	0.0007	*ABLIM1*
7	30090927-30155133	0.30	0.88	3.77	0.0033	*F1NF72*
7	31374271-31418061	0.42	1.00	2.88	0.0049	*LYPD1*, *NCKAP5*
7	33795201-33904515	0.47	1.00	2.20	0.0025	***LRP1B***
7	36818722-36875768	0.26	0.99	3.95	0.0024	*Q9DEH4*
7	37031922-37124566	0.56	1.00	2.92	0.0047	*STAM2*, *FMNL2*
8	5597-492518	0.56	0.99	1.94	0.0036	*F1NF53*
8	2178258-2252969	0.47	0.92	4.20	0.0004	*NEK7*
9	2952291-3007034	0.41	0.71	3.85	0.0044	/
10	763998-831991	0.50	0.78	5.04	0.0004	***MYO9A***, *F1P0M4*
11	9804894-9826761	0.47	0.52	3.11	0.0014	/
11	16253047-16303345	0.58	0.35	1.80	0.0040	/
12	1157199-1170169	0.37	0.63	4.57	0.0030	/
13	1533552-1640154	0.40	1.00	3.41	0.0049	*SRA1*, *APBB3*, *F1NH59*
14	8048059-8173629	0.42	0.86	3.09	0.0015	/
15	8495796-8543001	0.29	1.00	4.86	0.0003	*TBX6*, *CRKL*, *KLHL22*
15	8495796-8543001	0.29	0.99	4.43	0.0006	*TBX6*, *CRKL*, *KLHL22*
17	3250605-3271593	0.27	0.97	3.91	0.0033	/
17	4062173-4087131	0.26	0.94	3.77	0.0040	*C4PCF3*
18	4433126-4445816	0.26	0.79	5.99	0.0037	***PRPSAP1***
18	8365846-8400245	0.47	0.80	3.53	0.0038	/
23	935267-970086	0.31	0.91	5.40	0.0040	*EDN2*
24	5613517-5633477	0.28	0.86	4.38	0.0024	*ZW10*, *F1NC10*
24	6145308-6158962	0.31	0.80	5.30	0.0047	/

^1^REHH and *P*-values are presented for upstream and downstream sides from each core haplotype, respectively.

**Table 4:** Haplotype frequencies in the lean and fat lines of the core regions including 10 important genes

**Gene and core regions**	**Haplotype Number**	**Haploptypes**	**Haplotype frequency**		***P*-value^1^**
			**Lean line**	**Fat line**	
***MAOB***, ***MAOA***	1	CAAGG	0.645	0.615	<0.001
Chr1: 114789487-114875623	2	AAAGA	**0.197**	0	
	3	CGGAG	0.158	**0.269**	
	4	CGAGA	0	0.077	
	5	AAAGG	0	0.038	
***RB1***	1	GGAA	0.421	0.410	<0.001
Chr1: 173098805-173190831	2	GAGG	**0.368**	0.103	
	3	GAAA	0.211	0.192	
	4	AAGG	0	**0.244**	
	5	AGGA	0	0.038	
	6	GAGA	0	0.013	
***MYO7A***	1	AGG	**0.618**	0.090	<0.001
Chr1: 198071099-198113519	2	GAA	0.316	0.207	
	3	GGA	0.066	**0.652**	
	4	GAG	0	0.037	
	5	GGG	0	0.014	
***EHBP1***	1	GGG	**0.855**	0.090	<0.001
Chr3: 9177907-9222825	2	GAG	0.132	0.359	
	3	AGG	0.013	0.128	
	4	GGA	0	**0.423**	
***LRP2BP***	1	GGGG	0.443	0.487	<0.001
Chr4: 40653593-40713404	2	AAAA	**0.338**	0.211	
	3	GGAA	0.176	0	
	4	AAGG	0.044	**0.303**	
***BBS7***	1	AGGC	**0.605**	0.282	<0.001
Chr4: 55424480-55472209	2	GAAA	0.368	0.301	
	3	AAAA	0.026	0	
	4	AGAC	0	**0.198**	
	5	AGAA	0	0.161	
	6	GAAC	0	0.058	
***LRP1B***	1	AGAGAC	**0.361**	0.013	<0.001
Chr7: 33795201-33904515	2	GGAGGA	0.197		
	3	AGAAGA	0.105	0.154	
	4	GGGGGA	0.066	**0.449**	
	5	AGAAGC	0.057	0.346	
	6	GAGGGA	0.055	0.038	
	7	GAGAGA	0.050	0	
	8	GGAAGA	0.049	0	
	9	GAGGAA	0.026	0	
	10	GGAGAA	0.018	0	
	11	AGAAAC	0.016	0	
***MYO9A***	1	GGGAA	**0.355**	0.051	<0.001
Chr10: 763998-831991	2	AAGAA	0.276	0.358	
	3	AAGAG	0.237	0	
	4	GGGGA	0.118	0.013	
	5	AGGAA	0.013	0.065	
	6	AGAAA	0	**0.500**	
	7	AAAAA	0	0.013	
***PRPSAP1***	1	AGA	0.816	0.615	<0.001
Chr18: 4433126-4445816					
	2	GGG	**0.118**	0.026	
	3	AAG	0.066	0.090	
	4	AGG	0	**0.269**	

^1^*P*-values of Fisher’s Exact Test for difference analysis of haplotype frequencies between lean and fat lines.

### Mapping selection signatures to QTLs

The chicken QTL database available online (http://www.animalgenome.org/cgi-bin/QTLdb/GG/index) was explored to identify any overlapping of the core regions with significant REHH *P*-values (*P*<0.01) and published QTLs in chickens. The approximate positions of the overlapping QTLs for each core region are listed in Table 5. There were many overlaps between the core regions with significant REHH *P*-values (*P*<0.01) and published QTLs for AF content in chickens.

**Table 5:** **Reported QTL near the core regions with *P *<0.01 in the lean and fat lines**

**Lean line**						
Chr	Core region (bp)	Trait	QTL position (bp)	F-ratio	*P*-value	Reference
1	39360501-39455853	AFP	1937738-52700434	1.474	Suggestive	[21]
1	49926970-49964278	AFP	25998723-65961966	1.732	Suggestive	[21]
		AFW	25998723-65961966	1.882	Suggestive	[21]
		AFW	48175152- 51977642	8.14	Significant	[22]
1	173098805-173190831	AFW	158352237- 182910620	3.18	Significant	[23]
		AFP	171224834- 174526878	20.34	Significant	[23]
2	3631683-3739002	AFW	3097660- 4097660	3.38	Suggestive	[24]
3	3794973-3861882	AFP	800029-110574691	1.364	Suggestive	[21]
3	10257926-10454969	AFP	800029-110574691	1.364	Suggestive	[21]
		AFW	6841859-13986734	8.16	Significant	[22]
		AFP	6841859- 57396057	7.9	Significant	[25]
		AFW	6841859- 44850897	7.4	Significant	[25]
3	14895290-14957057	AFP	800029-110574691	1.364	Suggestive	[21]
		AFW	6841859-13986734	8.16	Significant	[22]
		AFP	6841859- 57396057	7.9	Significant	[25]
		AFW	6841859- 44850897	7.4	Significant	[25]
		AFW	13986734-25508863	\	Suggestive	[26]
3	26957549-26996618	AFP	800029-110574691	1.364	Suggestive	[21]
		AFP	6841859- 57396057	7.9	Significant	[25]
		AFW	6841859- 44850897	7.4	Significant	[25]
		AFW	24160710-51592221	\	Suggestive	[27]
		AFW	25508863- 35512024	\	Suggestive	[26]
3	27303800-27335510	AFP	800029-110574691	1.364	Suggestive	[21]
		AFP	6841859- 57396057	7.9	Significant	[25]
		AFW	6841859- 44850897	7.4	Significant	[25]
		AFW	24160710-51592221	\	Suggestive	[27]
		AFW	25508863- 35512024	\	Suggestive	[26]
3	27382993-27430067	AFP	800029-110574691	1.364	Suggestive	[21]
		AFP	6841859- 57396057	7.9	Significant	[25]
		AFW	6841859- 44850897	7.4	Significant	[25]
		AFW	24160710-51592221	\	Suggestive	[27]
		AFW	25508863- 35512024	\	Suggestive	[26]
3	35555718-35610466	AFP	800029-110574691	1.364	Suggestive	[21]
		AFW	35512024-40755790	18.5	Significant	[28]
		AFP	35512024-40755790	13.1	Significant	[28]
4	17765695-17819334	AFW	17425871-18425871	\	Significant	[29]
4	46149116-46190279	AFW	42005559- 51609571	2.26	Suggestive	[30]
4	55424480-55472209	AFP	51266614- 88408499	16.0	Significant	[25]
4	83051637-83117974	AFP	51266614- 88408499	16.0	Significant	[25]
		AFW	80258156-88408499	6.9	Significant	[25]
		AFW	81539616- 84618310	2.04	Suggestive	[30]
5	23825115-23872187	AFW	18412554-42717839	21.8	Significant	[25]
		AFP	18723157- 43339045	19.4	Significant	[25]
		AFW	19782191- 30162990	\	Suggestive	[26]
		AFW	19782191- 30162990	7.04	Significant	[31]
5	42592517-42679460	AFW	18412554-42717839	21.8	Significant	[25]
		AFP	18723157- 43339045	19.4	Significant	[25]
		AFW	37226264-53779276	6.74	Significant	[31]
6	35354459-35390346	AFP	29647151- 37399694	6.9	Significant	[25]
7	28869664-28906344	AFW	25306930- 38010856	\	Suggestive	[27]
		AFW	28166221- 29166221	9.78	Significant	[32]
		AFW	28166221- 29166221	\	Significant	[33]
7	35674098-35715122	AFW	25306930- 38010856	\	Suggestive	[27]
9	16264832-16366749	AFW	13658592-23770679	5.03	Suggestive	[22]
		AFW	15457880-16457880	7.0	Suggestive	[34]
10	19717086-19745274	AFP	16519830- 20778533	9.9	Significant	[28]
13	16758621-16783127	AFW	16327806- 18173123	2.10	Suggestive	[30]
15	7345639-7377799	AFW	1917251- 10769106	10.2	Significant	[25]
		AFP	2388961-10769106	12.8	Significant	[25]
		AFW	2798507-10769106	8.13	Significant	[22]
		AFW	2798507-10769106	5.67	Suggestive	[22]
		AFW	3717446-7928397	2.21	Suggestive	[30]
		AFP	3717446-7928397	2.22	Suggestive	[30]
Fat line						
1	51248496-51279543	AFP	1937738-52700434	1.474	Suggestive	[21]
		AFW	48175152- 51977642	8.14	Significant	[22]
		AFP	25998723- 65961966	1.732	Suggestive	[21]
		AFW	25998723- 65961966	1.882	Suggestive	[21]
1	58120009-58215364	AFP	25998723- 65961966	1.732	Suggestive	[21]
		AFW	25998723- 65961966	1.882	Suggestive	[21]
		AFW	55261695-67128747	12.18	Significant	[35]
1	60171076-60254771	AFP	25998723- 65961966	1.732	Suggestive	[21]
		AFW	25998723- 65961966	1.882	Suggestive	[21]
		AFW	55261695-67128747	12.18	Significant	[35]
1	67763862-67830818	AFW	67327367-68327367	\	Significant	[33]
1	68213617-68257241	AFW	67327367-68327367	\	Significant	[33]
1	101535615-101635667	AFW	89938943-167462479	9.4	Significant	[36]
		AFW	94157976- 102460326	6.11	Suggestive	[35]
1	114789487-114875623	AFW	113344161- 132660888	7.90	Suggestive	[35]
		AFW	114143603- 115143603	7.1	Significant	[36]
1	125909995-126011984	AFW	113344161- 132660888	7.90	Suggestive	[35]
1	181800227-181883545	AFW	158352237-182910620	3.18	Significant	[23]
3	9177907-9222825	AFP	800029- 110574691	1.364	Suggestive	[21]
		AFW	6841859- 13986734	8.16	Significant	[22]
		AFW	6841859- 13986734	5.8	Suggestive	[22]
		AFP	6841859-57396057	7.9	Significant	[25]
		AFW	6841859-44850897	7.4	Significant	[25]
3	16143474-16194865	AFP	800029- 110574691	1.364	Suggestive	[21]
		AFP	6841859-57396057	7.9	Significant	[25]
		AFW	6841859-44850897	7.4	Significant	[25]
		AFW	13986734-25508863	\	Suggestive	[26]
3	24945839-24986772	AFP	800029- 110574691	1.364	Suggestive	[21]
		AFP	6841859-57396057	7.9	Significant	[25]
		AFW	6841859-44850897	7.4	Significant	[25]
		AFW	13986734-25508863	\	Suggestive	[26]
		AFW	24160710-51592221	\	Suggestive	[27]
3	44265116-44311493	AFP	800029- 110574691	1.364	Suggestive	[21]
		AFP	6841859-57396057	7.9	Significant	[25]
		AFW	6841859-44850897	7.4	Significant	[25]
		AFW	24160710-51592221	\	Suggestive	[27]
		AFW	40755790-45203763	7.5	Significant	[28]
		AFP	40755790-45203763	10.8	Significant	[28]
3	69863850-69906698	AFP	800029- 110574691	1.364	Suggestive	[21]
3	85874137-85931473	AFP	800029- 110574691	1.364	Suggestive	[21]
3	97227680-97337906	AFP	800029- 110574691	1.364	Suggestive	[21]
4	40653593-40713404	AFP	40473174-41473174	\	Significant	[32]
4	55950677-55991394	AFP	51266614- 88408499	16.0	Significant	[25]
4	86719441-86754976	AFP	51266614- 88408499	16.0	Significant	[25]
		AFW	80258156-88408499	6.9	Significant	[25]
5	40239840-40261525	AFW	18412554- 42717839	21.8	Significant	[25]
		AFP	18723157- 43339045	19.4	Significant	[25]
		AFW	37226264- 53779276	6.74	Significant	[31]
		AFW	40158255- 41158255	\	Significant	[37]
		AFW	40158255- 41158255	\	Significant	[38]
		AFP	40158255- 41158255	\	Significant	[38]
5	47240577-47282933	AFW	37226264- 53779276	6.74	Significant	[31]
5	59811459-59880511	AFW	51748760-60234891	\	Significant	[26]
		AFW	53867807-62098509	11.87	Significant	[31]
		AFW	53867807-62098509	6.82	Significant	[31]
7	30090927-30155133	AFW	25306930- 38010856	\	Suggestive	[27]
7	31374271-31418061	AFW	25306930- 38010856	\	Suggestive	[27]
7	33795201-33904515	AFW	25306930- 38010856	\	Suggestive	[27]
		AFW	32440861-34526547	2.08	Suggestive	[30]
7	36818722-36875768	AFW	25306930- 38010856	\	Suggestive	[27]
7	37031922-37124566	AFW	25306930- 38010856	\	Suggestive	[27]
9	2952291-3007034	AFW	2798942-3798942	\	Significant	[32]
		AFP	2972071-3972071	\	Significant	[32]
11	9804894-9826761	AFW	6272742- 12810705	2.15	Suggestive	[30]
12	1157199-1170169	AFP	734209- 12275026	5.22	Significant	[28]
		AFP	734209- 12275026	4.51	Significant	[28]
		AFP	813709-1813709	\	Significant	[32]
15	8495796-8543001	AFW	1917251- 10769106	10.2	Significant	[25]
		AFP	2388961- 10769106	12.8	Significant	[25]
		AFW	2798507- 10769106	8.13	Significant	[22]
		AFW	2798507- 10769106	5.67	Suggestive	[22]
23	935267-970086	AFW	74802-1074802	\	Significant	[39]

## Discussion

Selective sweep is used to detect genomic regions with reduced variation in allele frequency in any population experiencing divergent selection for specific traits. Here, we determined the feasibility of the selective sweep approach for finding genes important for AF deposition in chickens. The long-range haplotype test was employed, which detects selection signature by measuring the characteristics of haplotypes within the lean and fat lines divergently selected for AF content. There were 5357 and 5593 core regions in the lean and fat lines, respectively. When comparing the average marker spacing with mean core length and number of SNPs forming cores, we revealed that core regions are more likely to appear in regions with higher marker density.

The selection signatures on the whole genome were calculated, and a subset of putative core regions with significant REHH *P*-values (*P*<0.01) was identified. The genes in these core regions were detected and 10 genes, including *RB1*, *BBS7*, *MAOA*, *MAOB*, *EHBP1*, *LRP2BP*, *LRP1B*, *MYO7A*, *MYO9A* and *PRPSAP1*, were important for fatness. Among these 10 important genes, seven genes, including *RB1*, *BBS7*, *MAOA*, *MAOB*, *EHBP1*, *LRP2BP* and *LRP1B*, were all in the QTL regions reported previously for AF in chickens (Table 5). Although the other three genes, including *MYO7A*, *MYO9A* and *PRPSAP1*, were not in the QTL regions, these genes were also important for the AF deposition.

The known functions of these 10 genes were analyzed and the results indicated that they were likely to be linked with fatness. The *RB1* gene regulates the C/EBP-DNA-binding activity during 3T3-L1 adipogenesis and plays a key role in adipocyte differentiation [40,41].

The *BBS7* gene is a member of the Bardet-Biedl syndrome (BBS) family. BBS is a pleiotropic genetic disorder characterized by obesity, photoreceptor degeneration, polydactyly, hypogenitalism, renal abnormalities, and developmental delay [42]. BBS is recognized to be a genetically heterogeneous autosomal recessive disorder mapped to eight loci [42]. Positional cloning and candidate genes identified six BBS genes, including *BBS1*, *BBS2*, *BBS4*, *BBS6*, *BBS7*, and *BBS8*[42]. These BBS genes may be important for obesity.

The *MAOA* and *MAOB* are two enzymes important for dopamine production. The dopamine levels influence the risk of obesity and *MAOA* and *MOAB* may be implicated in human obesity [43].

The *EHBP1* gene is required for insulin-stimulated GLUT4 movements [44]. Insulin stimulates glucose transport in adipose tissues by recruiting intracellular membrane vesicles containing the glucose transporter *GLUT4* to the plasma membrane [44]. The mechanisms involved in the biogenesis of these vesicles and their translocation to the cell surface were studied and the results indicated that *EHD1* and *EHBP1* are required for perinuclear localization of *GLUT4*, and the loss of *EHBP1* disrupts insulin-regulated *GLUT4* recycling in cultured adipocytes [44]. This indicates that the *EHBP1* gene may be important in adipocyte differentiation.

The *LRP2BP* and *LRP1B* genes are two members of the low-density lipoprotein receptor family that participates in a wide range of physiological processes, including the regulation of lipid metabolism, protection against atherosclerosis, neurodevelopment, and transport of nutrients and vitamins [45].

The *MYO7A* and *MYO9A* are two myosin genes. A spontaneous mutant mouse line, Myo7^ash1-6J^, was used to study the function of the *MYO7A* gene, and the result indicated that the mutant male homozygous mice displayed decreased body weight and body fat [46]. The *MYO9A* gene was in the BBS4 region of chromosome 15q22-q23 [47], which might be important for obesity.

The *PRPSAP1* gene is named as phosphoribosyl pyrophosphate synthetase-associated protein 1. The results of differentially expressed genes associated with insulin resistance indicate that *PRPSAP1* gene is associated with percentage of body fat [48].

The associations of these 10 genes with obesity or lipid metabolism were mainly in humans and mice. Because of the high conservation of these genes between humans, mice and chickens, the 10 genes might also be important for AF deposition in chickens.

## Conclusions

Our results provide a genome-wide map of selection signatures in two chicken lines divergently selected for AF content. There were 51 and 57 core regions showing significant *P*-values (*P*<0.01) of selection signatures in the lean and fat lines, respectively. In these core regions there were a number of important genes, including *RB1*, *BBS7*, *MAOA*, *MAOB*, *EHBP1*, *LRP2BP*, *LRP1B*, *MYO7A*, *MYO9A* and *PRPSAP1*. These genes are important for AF deposition in chickens.

## Abbreviations

AF: Abdominal fat; AFP: Abdominal fat percentage; AFW: Abdominal fat weight; BBS: Bardet-Biedl syndrome; CH: Core haplotypes; CR: Core region; EHH: Extended haplotype homozygosity; IHS: Integrated Haplotype Score; LD: Linkage disequilibrium; NEAU: Northeast Agricultural University; NEAUHLF: Northeast Agricultural University broiler lines divergently selected for abdominal fat content; NRC: National Research Council; QTL: Quantitative trait loci; REHH: Relative Extended Haplotype Homozygosity; SNP: Single nucleotide polymorphism; VLDL: Very low-density lipoprotein.

## Competing interests

There are no potential competing interests related to this manuscript.

## Authors’ contributions

HZ contributed to, conceived and designed the experiments, participated in the interpretation of the data, and drafted and wrote the manuscript. SZW participated in the design of the study and interpretation of the data, and contributed to writing the manuscript. ZPW participated in the design of the study and contributed to writing the manuscript. YD carried out analysis and interpretation of the data. NW participated in the design of the study and contributed to the analysis of the data. XXH participated in the analysis and interpretation of the data. YDZ participated in the design of the study and contributed to the analysis of the data. YXW participated in the design of the study. LL contributed reagents/materials/analysis tools. ZQT contributed reagents/materials/analysis tools. HL co-led the conception and design of the study, participated in the interpretation of the data, and contributed to writing the manuscript. All authors submitted comments on drafts, and read and approved the final manuscript.
